# Macroscopic complexity from an autonomous network of networks of theta neurons

**DOI:** 10.3389/fncom.2014.00145

**Published:** 2014-11-18

**Authors:** Tanushree B. Luke, Ernest Barreto, Paul So

**Affiliations:** School of Physics, Astronomy, and Computational Sciences and The Krasnow Institute for Advanced Study, George Mason UniversityFairfax, VA, USA

**Keywords:** theta neuron, type-I neuron, hierarchical network, neural field, macroscopic behavior, coherence, synchrony, chaos

## Abstract

We examine the emergence of collective dynamical structures and complexity in a network of interacting populations of neuronal oscillators. Each population consists of a heterogeneous collection of globally-coupled theta neurons, which are a canonical representation of Type-1 neurons. For simplicity, the populations are arranged in a fully autonomous driver-response configuration, and we obtain a full description of the asymptotic macroscopic dynamics of this network. We find that the collective macroscopic behavior of the response population can exhibit equilibrium and limit cycle states, multistability, quasiperiodicity, and chaos, and we obtain detailed bifurcation diagrams that clarify the transitions between these macrostates. Furthermore, we show that despite the complexity that emerges, it is possible to understand the complicated dynamical structure of this system by building on the understanding of the collective behavior of a single population of theta neurons. This work is a first step in the construction of a mathematically-tractable network-of-networks representation of neuronal network dynamics.

## 1. Introduction

The brain is a complex hierarchical network of networks (Zhou et al., 2006; Bullmore and Sporns, 2009; Meunier et al., 2010). Neurons are organized into different neuronal assemblies, and these neuronal assemblies interact with each other, forming larger assemblies (Sherrington, 1906; Hebb, 1949; Harris, 2005). But while there is a wealth of knowledge on the microscopic scale regarding the dynamics of individual neurons, the macroscopic behavior of such interacting populations of neurons is not well understood. Indeed, the functional and information-processing activity of the brain, from perception to consciousness, is thought to result from the emergent collective behavior of these assemblies.

In recent years, the mathematical study of networks of this kind, based on globally-coupled populations of simple phase oscillators, has advanced significantly. This is in large part due to new analytical techniques (Ott and Antonsen, 2008, 2009; Marvel et al., 2009; Ott et al., 2011; Pikovsky and Rosenblum, 2011). These techniques enable the derivation of low-dimensional dynamical systems that reveal the collective emergent behavior of the full discrete population (in the limit of an infinite number of interacting elements). In the context of computational neuroscience, these methods were applied to autonomous globally-coupled networks of canonical Type-I neurons (i.e., theta neurons) by Luke et al. (2013), and to non-autonomous theta neuron networks by So et al. (2014). More recently, Laing (2014) extended these results to include space-dependent coupling. A similar approach, based on phase-response curves, was pursued by Pazó and Montbrió (2014).

Of course, such networks lack the intricate connectivity found in real biological networks. Nevertheless, they are ideal building blocks for the construction of a more realistic, yet mathematically tractable, network-of-networks representation of the brain. In the current study, we consider the simplest hierarchical structure as a first step in this process. Using two globally-coupled networks of theta neurons, we arrange for the activity of one population to drive the second population. Thus, the overall network has an autonomous driver-response configuration. We demonstrate that even in this simplest network-of-networks, the collective behavior of the response network can exhibit a full range of complex behavior, from simple collective rhythms to temporally chaotic dynamics. Most importantly, we provide a complete non-linear dynamical analysis of this system, including predictive bifurcation diagrams for the behavior of the response population in terms of the driver's dynamics and the network characteristics.

## 2. Recap of single population results

### 2.1. The theta neuron

Neurons are typically classified into two types, based on the nature of the onset of spiking as a constant injected current exceeds an effective threshold (Hodgkin, 1948; Ermentrout, 1996; Izhikevich, 2007). Type-I neurons begin to spike at an arbitrarily low rate, whereas Type-II neurons spike at a non-zero rate as soon as the threshold is exceeded. Neurophysiologically, excitatory pyramidal neurons are often of Type-I, and fast-spiking inhibitory interneurons are often of Type-II (Nowak et al., 2003; Tateno et al., 2004). Near the onset of spiking, Type-I neurons can be represented by a canonical phase model that features a saddle-node bifurcation on an invariant cycle, or SNIC bifurcation (Ermentrout and Kopell, 1986; Ermentrout, 1996). This model has come to be known as the theta neuron, and is given by

(1) $$\dot{\theta }=(1-\cos\theta )+(1+\cos\theta )\eta ,$$

where θ is a phase variable on the unit circle and η is a bifurcation parameter related to the injected current. For η < 0, the neuron is attracted to a stable equilibrium which represents the resting state. An unstable equilibrium is also present, representing the threshold. If an external stimulus pushes the neuron's phase across the unstable equilibrium, θ will move around the circle and approach the resting equilibrium from the other side. When θ crosses θ = π, the neuron is said to have spiked. Thus, for η < 0, the neuron is excitable. As the parameter η increases, these equilibria approach each other and merge via the SNIC bifurcation at η = 0. At this point, the equilibria disappear, leaving a limit cycle. The neuron spikes regularly for η > 0. In the following, we call η the “excitability parameter.”

### 2.2. A network of theta neurons

We formulate a single population of *N* theta neurons as follows:

(2) $$\dot{\theta_{j}}=\left(1-\cos\theta_{j}\right)+\left(1+\cos\theta_{j}\right)\left[\eta_{j}+I_{syn}\right],$$

where *j* = 1, …, *N* is the index for the *j*-th neuron. The neurons are coupled via a pulse-like synaptic current

(3) $$I_{syn}=\frac{k}{N}\sum_{i\;=\;1}^{N}P_{n}(\theta_{i}),$$

where *P_n_*(θ) = *a_n_* (1 − cosθ)^*n*^, *n* ∈ ℕ, and *a_n_* is a normalization constant^1^ such that

$$\int_{0}^{2\pi }P_{n}(\theta )d\theta =2\pi .$$

The parameter *n* defines the sharpness of the pulse-like synapse in that *P_n_*(θ) becomes more and more sharply peaked as *n* increases. We assume that the synaptic strength *k* is the same for all neurons.

Note that the connectivity described by Equations (2) and (3) includes self-coupling terms. These have negligible effect on the collective network dynamics (data not shown), which is to be expected since they represent only one out of *N* inputs to any given neuron. Nevertheless, we note that these self-connections have real-world analogs in “autapses,” which have been found in several regions of the brain (e.g., Bacci et al., 2003; Bekkers, 2003).

Neurons in real biological networks exhibit a range of different intrinsic dynamics. We model this by taking the excitability parameter η_*j*_ of each neuron to be different, with each η*_j_* being drawn randomly from a distribution *g*(η). In the following analysis, we assume a Lorentzian distribution,

(4) $$\begin{array}{cc} g(\eta )=\frac{1}{\pi }\frac{\Delta }{{\left(\eta -\eta_{0}\right)}^{2}+\Delta^{2}}, & \left(4\right) \end{array}$$

where η_0_ is the center of the distribution, and Δ, the half-width at half-maximum, describes the degree of heterogeneity in the population.

### 2.3. Reduction and asymptotic states of the single population

The macroscopic behavior of our network can be quantified by the “macroscopic mean field,” or order parameter, defined as

(5) $$\tilde{z}(t)=\sum_{j\;=\;1}^{N}e^{i\theta_{j}},$$

where the tilde indicates that the sum is over a finite population of *N* oscillators. (Below we will drop the tilde in the case of an infinite network.) The magnitude of the order parameter |$\tilde{z}$(*t*)| ∈ [0, 1] quantifies the degree of synchronization present at time *t*.

In Luke et al. (2013), we used the Ott-Antonsen method (Ott and Antonsen, 2008, 2009; Ott et al., 2011) to derive a low-dimensional dynamical system whose asymptotic dynamics can be shown to coincide with that of the order parameter of the single-population network defined above (Equations 2–4), in the limit *N* → ∞. This reduced dynamical system is

(6) $$\dot{z}=-i\frac{{\left(z-1\right)}^{2}}{2}+\frac{{\left(z+1\right)}^{2}}{2}\left\{-\Delta +i\left[\eta_{0}+kH_{n}(z)\right]\right\},$$

where

(7) $$H_{n}(z)=I_{syn}/k=a_{n}\left(A_{0}+\sum_{q\;=\;1}^{n}A_{q}(z^{q}+z^{*q})\right),$$

(8) $$A_{q}=\sum_{j,m\;=\;0}^{n}\delta_{j-2m,q}Q_{jm},$$

and

(9) $$Q_{jm}=\frac{{\left(-1\right)}^{j\;-\;2m}n!}{2^{j}m!(n-j)!(j-m)!}.$$

In these equations, z^*^ denotes the complex conjugate of *z*, and δ_*i,j*_ is the Kronecker delta function on the indices (*i,j*). Note that *H_n_*(*z*) = H^*^_n_(*z*) is a real-valued function.

The analysis of Equations (6–9) reported in Luke et al. (2013) showed that the theta neuron network can exhibit three types of asymptotic states. These correspond to a node, a focus, and a limit cycle in the order parameter. A complete bifurcation analysis describing how these states change as the parameters *k*, η_0_, and Δ change was also reported. For our purposes in the current work, we now briefly describe the three possible collective macroscopic states.

We called the node, focus, and limit cycle solutions the “Partially Synchronous Rest” (PSR), “Partially Synchronous Spiking” (PSS), and “Collective Periodic Wave” (CPW) states, respectively. In the PSR state, most neurons remain at rest, while in the PSS state, most neurons spike continuously. Nevertheless, in both these states, the macroscopic mean field (or order parameter) sits at an equilibrium. In contrast, the CPW state corresponds to periodic oscillations of the complex order parameter, and typically, both |*z*(*t*)| and arg (*z*) oscillate in time indicating that the individual neurons clump together and spread apart in a periodic fashion. We refer the interested reader to Luke et al. (2013) for further details, including movies that illustrate both the microscopic and macroscopic behaviors of these collective states.

## 3. Formulation of the driver-response network

In this work, we are interested in the dynamics exhibited by a network of two coupled populations of theta neurons. We formulate the general case, but restrict analysis to the simplest such configuration: a driver-response network.

### 3.1. General two-population model

Extending the model described above, a general formulation of a pair of interacting populations of theta neurons can be expressed as follows:

(10) $$\begin{array}{l} {\dot{\theta }}_{1,j}=1+\eta_{1,j}-(1-\eta_{1,j})\cos\theta_{1,j}+a_{n}(1+\cos\theta_{1,j})\\ \;\left[\frac{k_{11}}{N_{1}}\sum_{p\;=\;1}^{N_{1}}{\left(1-\cos\theta_{1,p}\right)}^{n}+\frac{k_{12}}{N_{2}}\sum_{q\;=\;1}^{N_{2}}{\left(1-\cos\theta_{2,q}\right)}^{n}\right]\text{​​},\\ {\dot{\theta }}_{2,j}=1+\eta_{2,j}-(1-\eta_{2,j})\cos\theta_{2,j}+a_{n}(1+\cos\theta_{2,j})\\ \;\left[\frac{k_{21}}{N_{1}}\sum_{p\;=\;1}^{N_{1}}{\left(1-\cos\theta_{1,p}\right)}^{n}+\frac{k_{22}}{N_{2}}\sum_{q\;=\;1}^{N_{2}}{\left(1-\cos\theta_{2,q}\right)}^{n}\right]\text{​​}, \end{array}$$

where θ_1,*j*_ and θ_2,*j*_ denote the *j*th neuron in the first and second populations, respectively, and the extension to any number of interacting populations is straightforward. The excitability parameters η_1,*j*_ and η_2,*j*_ are randomly drawn from two independent Lorentzian distributions as in Equation (4), with medians η_1_, η_2_ and widths Δ_1_, Δ_2_, respectively. We take the sharpness parameter of the pulse-like synaptic interaction, *n*, to be the same for both populations. Macroscopic mean field parameters $\tilde{z}$_1_(*t*), $\tilde{z}$_2_(*t*) can be defined for each population by analogy with Equation (5).

Adapting the procedures described in Luke et al. (2013), we derived the Ott-Antonsen reduction of the coupled networks of Equation (10). This resulted in the following dynamical system:

(11) $$\begin{array}{l} {\dot{z}}_{1}=-i\frac{{\left(z_{1}-1\right)}^{2}}{2}+\frac{{\left(z_{1}+1\right)}^{2}}{2}\\ \;\left\{-\Delta_{1}+i\left[\eta_{1}+k_{11}H_{n}(z_{1})+k_{12}H_{n}(z_{2})\right]\right\},\\ {\dot{z}}_{2}=-i\frac{{\left(z_{2}-1\right)}^{2}}{2}+\frac{{\left(z_{2}+1\right)}^{2}}{2}\\ \;\left\{-\Delta_{2}+i\left[\eta_{2}+k_{21}H_{n}(z_{1})+k_{22}H_{n}(z_{2})\right]\right\}. \end{array}$$

with *H*_*n*_(*z*) defined as in Equations (7–9). As before, the asymptotic dynamics of Equation (11) can be shown to coincide with that of the order parameters of the populations in the network of Equation (10), in the limit *N*_1_, *N*_2_ → ∞.

We showed in Luke et al. (2013) that the dynamical structure of the single population depends rather weakly on the synaptic sharpness parameter *n*. Furthermore, we argued that a modest sharpness is more biophysically plausible than the δ-function coupling obtained in the limit *n* → ∞. Thus, from here on, we fix *n* = 2 and drop the subscript on *H_n_* to ease notation.

### 3.2. The driver-response system

**Figure 1:**
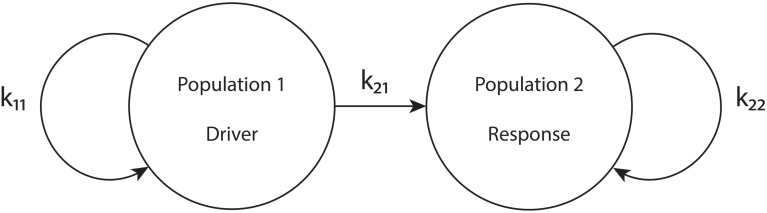
**The driver-response configuration**. *k*_11_ and *k*_22_ are the intra-population coupling strengths for populations 1 and 2, respectively, and *k*_21_ is the uni-directional coupling strength between the driver population (1) and the response population (2).

To put our network in the driver-response form, we set *k*_12_ = 0, so that population 1 receives no input from population 2. Therefore, the macrostates and bifurcations of population 1 are identical to those explored in Luke et al. (2013), described above. However, we allow *k*_21_ ≠ 0. Our goal is to examine the consequences of the influence of population 1 on population 2. We call population 1 the “driver” and population 2 the “response” system. See Figure 1.

Writing the governing equation of population 2 as

(12) $${\dot{z}}_{2}=-i\frac{{\left(z_{2}-1\right)}^{2}}{2}+\frac{{\left(z_{2}+1\right)}^{2}}{2}\left\{-\Delta_{2}+i\left[\eta_{eff}+k_{22}H(z_{2})\right]\right\}$$

with

(13) $$\eta_{eff}\equiv \eta_{2}+k_{21}H(z_{1}),$$

and comparing to Equation (6), we see that the behavior of population 2 is the same as that of a single population of theta neurons with an effective median excitability parameter η_*eff*_. This effective parameter depends on the median excitability parameter intrinsic to population 2 η_2_, the inter-population coupling *k*_21_, and the state of the driver *z*_1_.

Note that η_*eff*_ depends linearly on both η_2_ and *k*_21_ and non-linearly on the driver's state *z*_1_ through *H*(*z*_1_). Additionally, η_*eff*_ may be time-dependent if population 1 exhibits a CPW state, since in that case *z*_1_ oscillates periodically. In the following, we will examine all these cases.

## 4. Results

We will examine the behavior of population 2 as various parameters are varied. We organize the presentation of our results by first considering the case in which the driver population exhibits an equilibrium state. Later, we consider the case in which the driver population exhibits periodic behavior.

We will mainly consider two configurations of the response system. The “excitatorily coupled” response system has *k*_22_ > 0, and the “inhibitorily coupled” response system has *k*_22_ < 0. Other parameters are as noted below.

**Figure 2:**
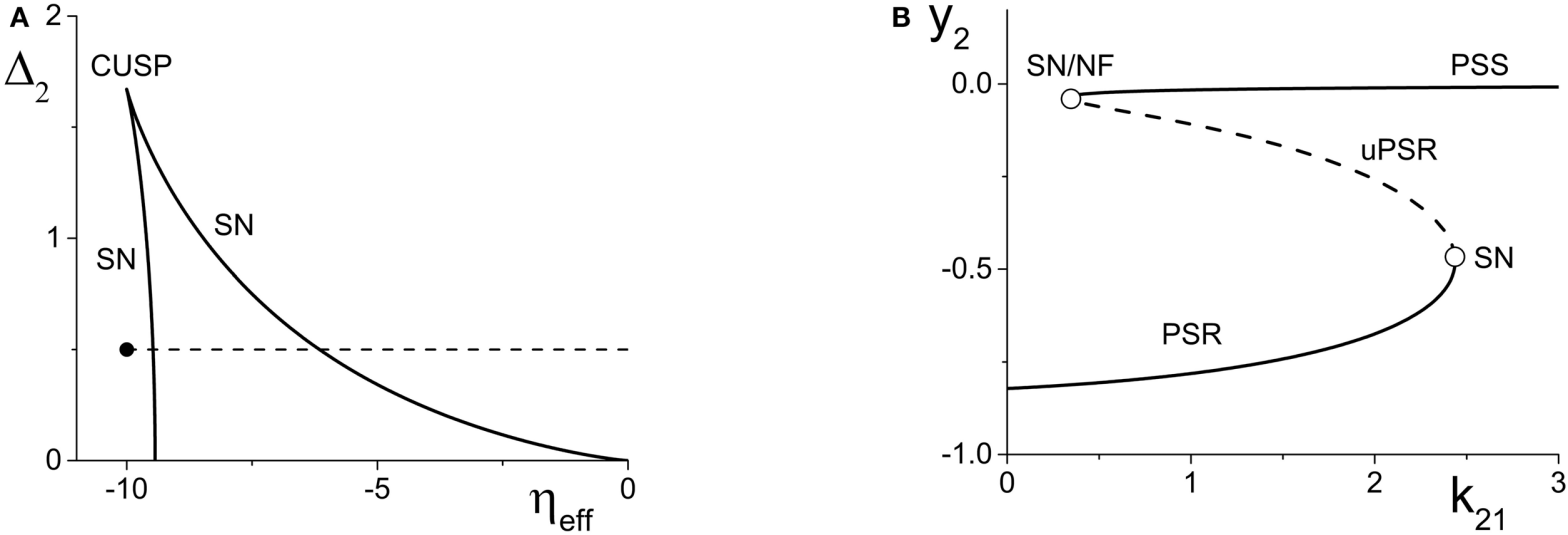
**(A)** A two-dimensional bifurcation diagram of the excitatorily-coupled response system. The heavy black lines are saddle-node (SN) bifurcation curves, and the solid dot denotes the parameters of the response system when decoupled from the driver. In the cases considered in the main text, the driver causes η_*eff*_ to vary along the horizontal dotted line. The parameters are: η_1_ = −0.2, Δ_1_ = 0.1, *k*_11_ = −2, and *k*_22_ = 9. **(B)** The one-dimensional bifurcation diagram showing the asymptotic values of *y*_2_ = Im(*z*_2_) vs. *k*_21_. Solid and dashed curves indicate stable and unstable equilibria, respectively, corresponding to partially synchronous spiking (PSS) and partially synchronous resting (PSR) states. The parameters are as in **(A)**, with η_2_ = −10 and Δ_2_ = 0.5.

**Figure 3:**
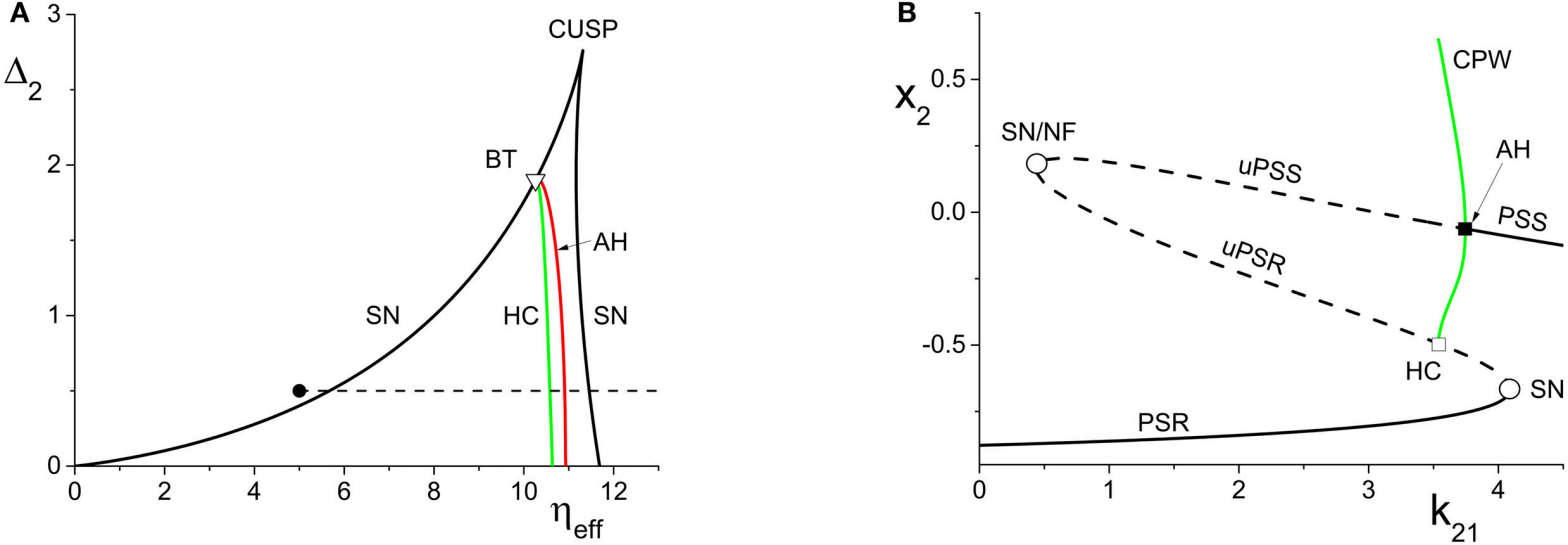
**(A)** The two-dimensional bifurcation diagram of the inhibitorily-coupled response system. The heavy black lines are saddle-node (SN) bifurcation curves, green is a homoclinic (HC) bifurcation curve, and red is an Andronov-Hopf (AH) bifurcation curve. The latter two curves emerge from a Bogdanov-Takens (BT) point. The solid dot denotes the parameters of the response system when decoupled from the driver. In the cases considered in the main text, the driver causes η_*eff*_ to vary along the horizontal dotted line. The parameters are: η_1_ = −0.2, Δ_1_ = 0.1, *k*_11_ = −2, and *k*_22_ = −9. **(B)** The one-dimensional bifurcation diagram showing the asymptotic value of *x*_2_ = Re(*z*_2_) vs. *k*_21_. Solid curves denote stable equilibria; dashed black curves are unstable equilibria. Green represents the maxima and minima of a collective periodic wave (CPW) limit cycle. The parameters are as in **(A)**, with η_2_ = 5 and Δ_2_ = 0.5.

**Figure 4:**
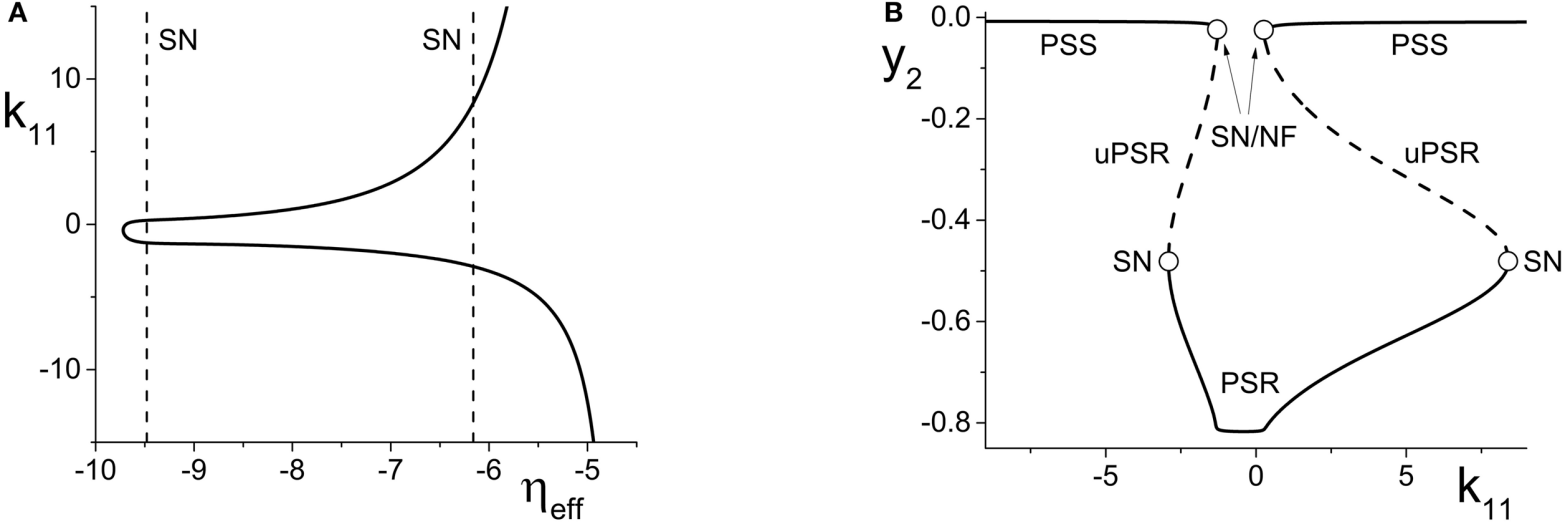
**(A)** The non-linear behavior of η_*eff*_ as a function of *k*_11_ for the excitatorily-coupled response system. η_*eff*_ is plotted horizontally to facilitate comparison with Figure 2A. The parameters are: η_1_ = −0.05, Δ_1_ = 0.05, η_2_ = −10, with the inter-population coupling fixed at *k*_21_ = 2.0. **(B)** The one-dimensional bifurcation diagram showing the asymptotic value of *y*_2_ = Im(*z*_2_) vs. *k*_11_. Solid and dashed curves indicate stable and unstable equilibria, respectively. The parameters are as in **(A)**, with Δ_2_ = 0.5 and *k*_22_ = 9.

**Figure 5:**
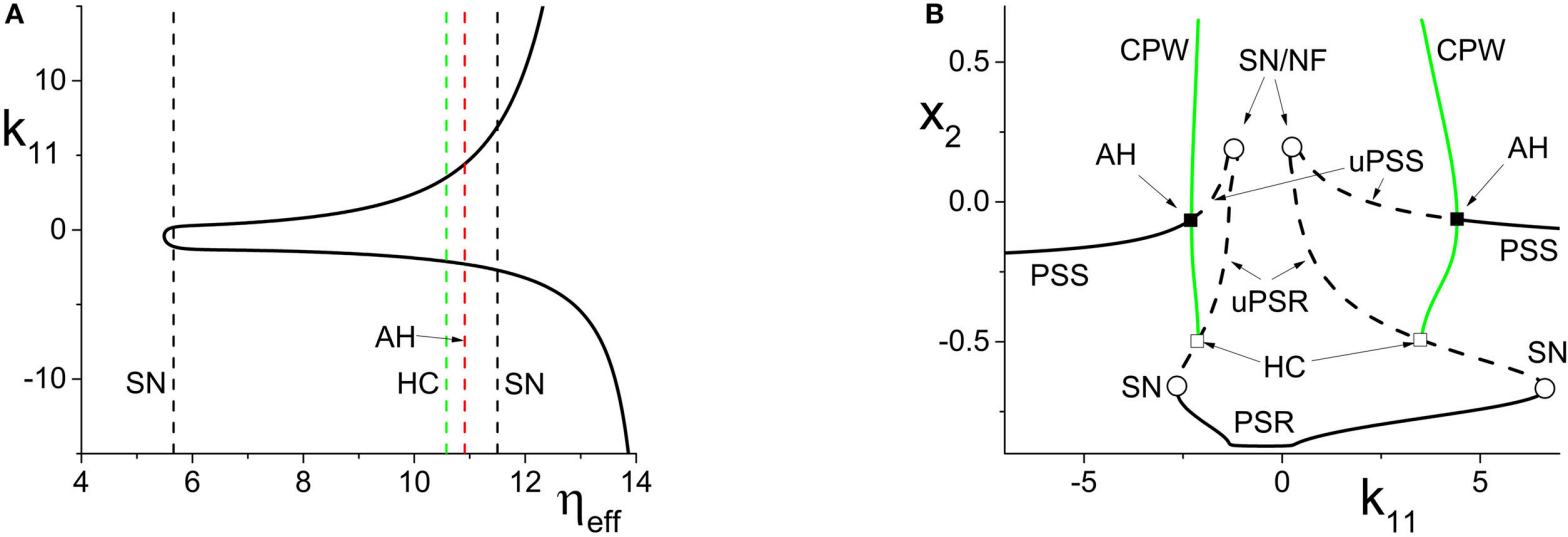
**(A)** The non-linear behavior of η_*eff*_ as a function of *k*_11_ for the inhibitorily-coupled response system. η_*eff*_ is plotted horizontally to facilitate comparison with Figure 3A. **(B)** The one-dimensional bifurcation diagram showing the asymptotic value of *x*_2_ = Im(*z*_2_) vs. *k*_11_. Solid and dashed black curves indicate stable and unstable equilibria, respectively, and green represents the maxima and minima of a CPW limit cycle state. The parameters are: η_1_ = −0.05, Δ_1_ = 0.05, η_2_ = 5, Δ_2_ = 0.5, and *k*_22_ = −9. The inter-population coupling is fixed at *k*_21_ = 3.5.

The bifurcation diagrams that appear below in Figures 2, 3, 4B, 5B, **8C** were obtained using XPPAUT (Ermentrout, 2002). Data for all other figures were generated using custom-designed code.

### 4.1. Driver on a macroscopic equilibrium

We begin by fixing the driving population's parameters at η_1_ = −0.2, Δ_1_ = 0.1, and *k*_11_ = −2, which corresponds to a PSR state. Thus, *z*_1_ remains fixed at a constant value. We examine the behavior of the two response system configurations as we vary the inter-population coupling parameter, *k*_21_. From Equation (13), η_*eff*_ varies linearly with respect to *k*_21_.

#### 4.1.1. Excitatorily-coupled response system

We set the response system's internal coupling to *k*_22_ = 9, and show in Figure 2A the two-parameter bifurcation diagram of the response system with respect to Δ_2_ and η_*eff*_. Two saddle-node bifurcation curves which meet at a cusp are seen. To the left of these curves, the response network exhibits a PSR state, and to the right, a PSS state. These states coexist inside the approximately triangular region.

We set the remaining parameters of the response system to η_2_ = −10 and Δ_2_ = 0.5. Thus, for *k*_21_ = 0, η_*eff*_ = η_2_, and the response system is situated at the solid black point marked in Figure 2A. As *k*_21_ increases from zero, η_*eff*_ increases linearly along the dotted line in Figure 2A, starting from the black point. In so doing, it traverses the SN bifurcation curves. Figure 2B shows how the imaginary part of the response's asymptotic macroscopic mean field [*y*_2_ = Im(*z*_2_)] changes with respect to *k*_21_, illustrating the coexistence of the stable PSR and PSS states, along with an unstable PSR state (uPSR).

The point marked “SN/NF” in Figure 2B indicates that as *k*_21_ increases, a saddle node bifurcation is encountered, corresponding to the left SN curve in Figure 2A. This creates a stable and an unstable PSS state. However, the unstable PSS state converts into an unstable PSR state at a value of *k*_21_ very slightly beyond the SN bifurcation. That is, the node corresponding to the unstable PSS state becomes a unstable PSR focus, a transition we called a Node-Focus (NF) transition in Luke et al. (2013). The distinction between these events is indistinguishable in the figure.

#### 4.1.2. Inhibitorily-coupled response system

We performed a similar analysis for the case in which the response system's internal coupling is *k*_22_ = −9, i.e., inhibitory, and η_2_ = 5. The remaining parameters were unchanged. The results are shown in Figure 3. In this case, the two-dimensional bifurcation diagram of the response system with respect to Δ_2_ and η_*eff*_ (Figure 3A) shows a similar (but mirror-image) cusp of saddle-node curves. A new feature is the occurrence of a codimension-2 Bogdanov-Takens (BT) point on the left SN curve, and the emergence of homoclinic (HC; green) and Andronov-Hopf (AH; red) bifurcation curves from the BT point.

Figure 3B shows how the real part of the response's asymptotic macroscopic mean field [*x*_2_ = Re(*z*_2_)] changes with respect to *k*_21_. As before, η_*eff*_ increases linearly as *k*_21_ increases, starting from the black solid point in Figure 3A and moving toward the right, traversing the various bifurcation curves along the dotted line. Note the presence of the attracting limit cycle CPW state in Figure 3B, which emerges at the HC bifurcation and terminates at the AH bifurcation as *k*_21_ increases.

It is interesting to note that in both cases described above, the same bifurcation structure would be encountered if, instead of varying *k*_21_ with a fixed value η_2_, we varied η_2_ with a fixed value of *k*_21_. While this is obvious from Equation (13) since *H*(z_1) is constant in these cases, this leads to the non-obvious conclusion that by modifying either the inter-population coupling or the intrinsic median excitability of the response population—two rather different system characteristics—one obtains identical transitions in the response network.

#### 4.1.3. Variation of the driver's macroscopic equilibrium

In the cases we considered previously, η_*eff*_ changed linearly with respect to the inter-population coupling *k*_21_. We now turn our attention to the effects incurred by altering the value of the driver influence function *H*(*z*_1_) in Equation (13). We do this by varying the driver's internal coupling strength *k*_11_, thus causing the driver's asymptotic macroscopic mean field *z*_1_ to change. This manipulation has the effect of changing η_*eff*_ *non-linearly* with respect to *k*_11_.

For simplicity, we only consider a range of *k*_11_ such that the driver always remains on a macroscopic equilibrium state, and we fix the inter-population coupling at *k*_21_ = 2.

We begin with the case of the excitatorily-coupled response system considered above, with η_2_ = −10, Δ_2_ = 0.5, and *k*_22_ = 9, and choose the remaining driver parameters to be η_1_ = −0.05 and Δ_1_ = 0.05. Figure 4A shows the non-linear behavior of η_*eff*_ as *k*_11_ is varied. Even though we are considering *k*_11_ to be the independent parameter, we plot η_*eff*_ horizontally so that it may be easily compared to Figure 2A; recall that this shows the two-dimensional bifurcation diagram of the response system. Now, as *k*_11_ changes, η_*eff*_ moves back and forth along the dotted line non-linearly. In particular, Figure 4A shows that for very negative values of *k*_11_, η_*eff*_ is near −5, which corresponds to a point in Figure 2A to the right of the SN curves. As *k*_11_ increases, η_*eff*_ decreases to approximately −10, thus crossing both SN curves in Figure 2A from right to left in the process. η_*eff*_ subsequently increases, and goes back across the SN curves from left to right. Note that Figure 4A includes vertical lines marking the position of the SN bifurcations (i.e., the values of η_*eff*_ at which the horizontal line at Δ_2_ = 0.5 in Figure 2A crosses the SN curves).

Figure 4B shows the behavior of the asymptotic state of the response system [*y*_2_ = Im(*z*_2_)] as a function of *k*_11_. This shows that as *k*_11_ increases, the response system passes through two separate regions of bistability, corresponding to the two traversals of the triangular bistable region in Figure 2A. Thus, Figure 4B is qualitatively similar to two copies of Figure 2B, with the structure for *k*_11_ < 0 reversed. Note that the two regions are not symmetrical. This is due to the non-symmetric behavior of η_*eff*_ as *k*_11_ changes.

Next, we examine how the same manipulation of the driver system affects the inhibitorily-coupled response system. The parameters are as above, but with η_2_ = 5 and *k*_22_ = −9. Figure 5A shows how η_*eff*_ changes as *k*_11_ is varied, again plotted with η_*eff*_ on the horizontal axis for ease of comparison with Figure 3A. Note the vertical lines in Figure 5A marking the SN, HC, and AH bifurcations.

The one-dimensional bifurcation diagram depicting the asymptotic state of the response system as a function of *k*_11_ is shown in Figure 5B. A situation similar to the previous case is observed. Two distorted versions of the structure of Figure 3B, with the features for *k*_11_ < 0 being reversed, are seen. Again, this is due to the non-linear and asymmetric behavior of η_*eff*_ as it traverses the bifurcations in Figure 3A twice: first right to left, and then left to right, as *k*_11_ is increased. Note also the presence of an attracting limit cycle CPW state in intervals of both positive and negative *k*_11_.

### 4.2. Driver on a macroscopic limit cycle

We now focus on the behavior of the response population when the driver is on a CPW state, which is a limit cycle of the driver's macroscopic mean field (or order parameter). Throughout this section, we fix the driver parameters at η_1_ = 10.75, *k*_11_ = −9, and Δ_1_ = 0.5, which results in a CPW driver state for which *H*(*z*_1_) oscillates periodically in time. In particular, we have *H*(*z*_1_) > 0 for all time. Thus, according to Equation (13), η_*eff*_ also oscillates periodically for *k*_21_ ≠ 0, and both the centroid and the amplitude of the η_*eff*_ oscillation increase as *k*_21_ increases.

We show below that in this configuration, the response population can exhibit periodic, multistable, chaotic, and/or quasiperiodic behavior, depending on the response system's parameters and the interpopulation coupling strength *k*_21_.

#### 4.2.1. Periodic behavior in the response system

**Figure 6:**
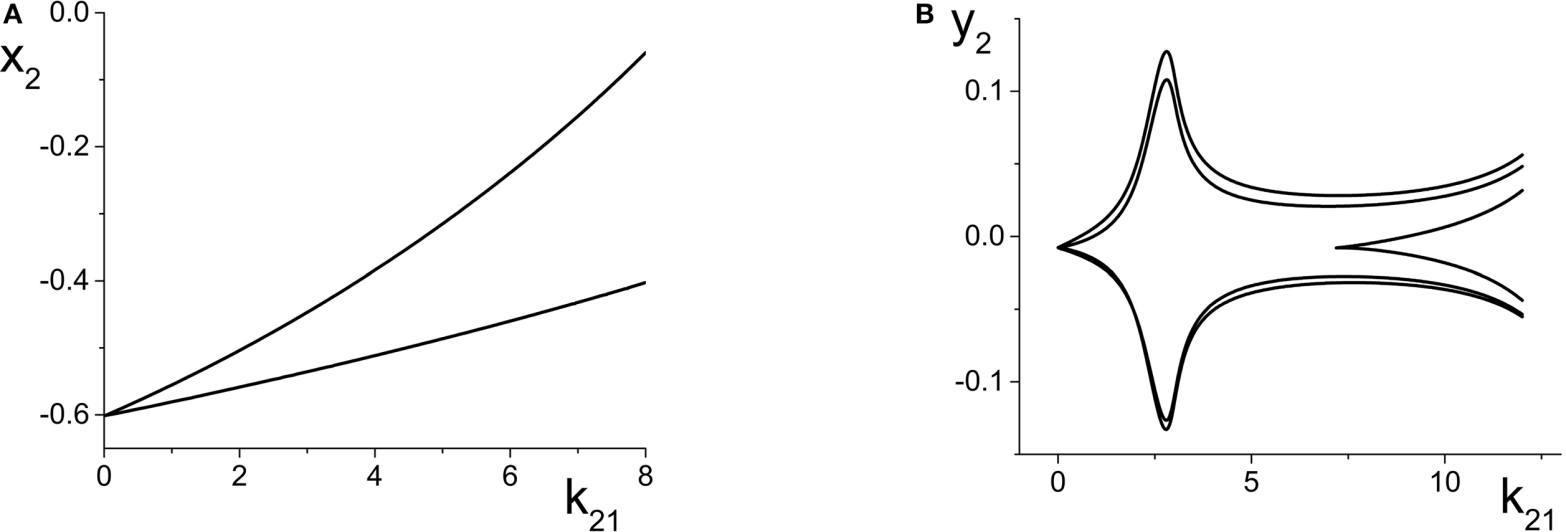
**(A)** Simple periodic behavior in the response system driven by a CPW state of the driver as a function of the inter-population coupling strength *k*_21_. The curves are local maxima and minima of *x*_2_ = Re(*z*_2_). The driver parameters are η_1_ = 10.75, Δ_1_ = 0.5, and *k*_11_ = −9, and the response parameters are η_2_ = −20, Δ_2_ = 0.5, and *k*_22_ = 9. **(B)** Slightly more complicated periodic behavior obtained at the same parameters, except with η_2_ = −5. The curves are local maxima and minima of *y*_2_ = Im(*z*_2_).

We begin by considering the excitatorily coupled response system, with Δ_2_ = 0.5 and *k*_22_ = 9, but with η_2_ = −20. When decoupled from the driver, this places the response system at a point well to the left in the parameter space of Figure 2A. Thus, the response system in isolation asymptotes to a PSR state. As *k*_21_ is increased from zero to eight, η_*eff*_ oscillates back and forth along the horizontal line in Figure 2A at Δ_2_ = 0.5, but always stays to the left of the SN curves shown in that figure. Thus, the driver simply pushes the response system's PSR state back and forth, avoiding any bifurcations. The result is simple periodic behavior in the driven response system. Figure 6A shows a plot of the maximum and minimum of *x*_2_ = Re(*z*_2_) vs. *k*_21_. As *k*_21_ increases, the amplitude of this simple periodic behavior increases. We observe that the frequency of the response system's oscillation is the same as that of the driver throughout this range of interpopulation coupling.

We now change the response system such that η_2_ = −5, and leave all other parameters the same as above. This change places the response system at a point to the right of the SN curves in Figure 2A, and for these parameters, the uncoupled response system asymptotes to a PSS state. Once again, as *k*_21_ increases, η_*eff*_ oscillates back and forth along the Δ_2_ = 0.5 line in Figure 2A, but this time it does so always staying to the right of the SN curves.

**Figure 7:**
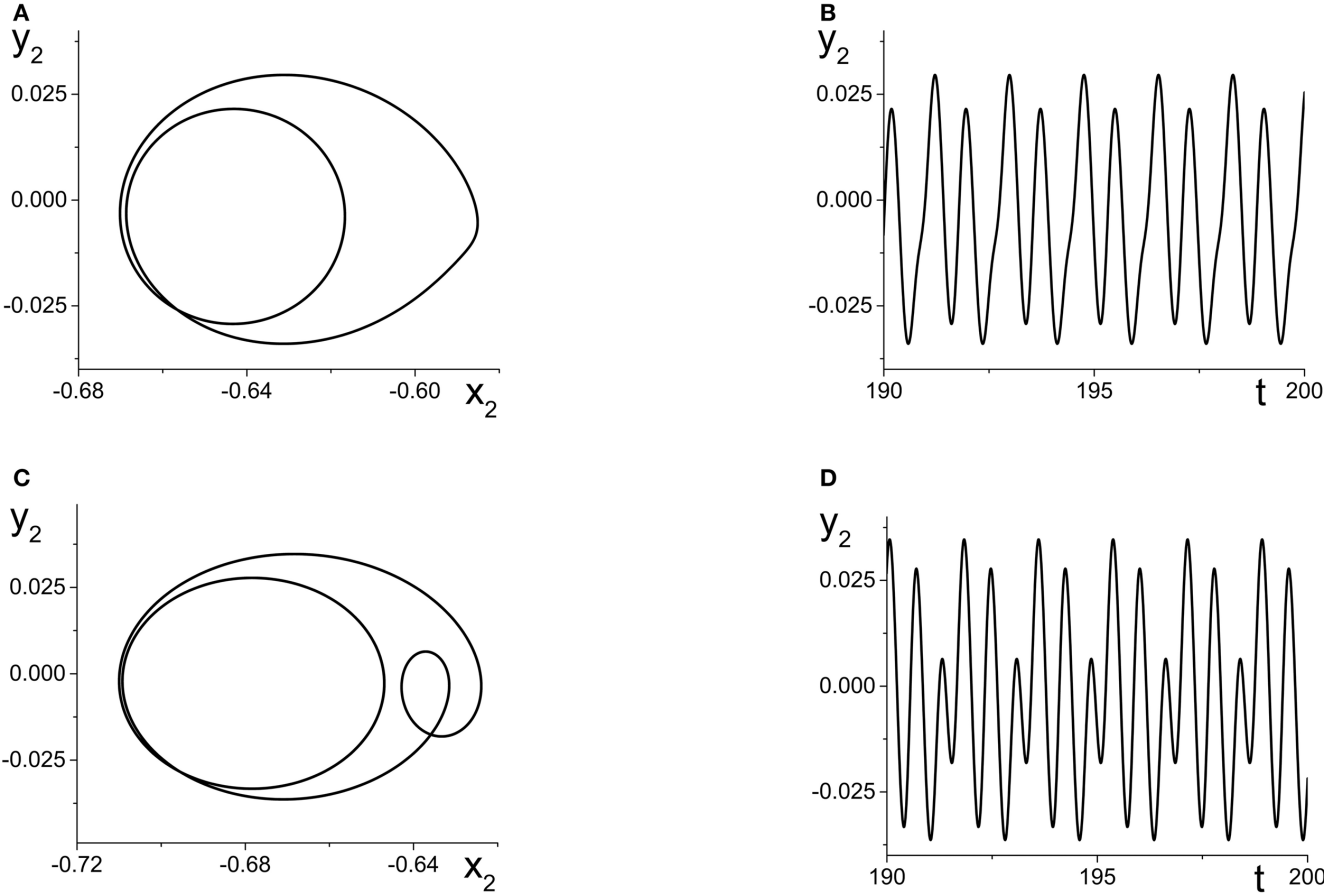
**The response system's behavior at parameters corresponding to Figure 6B at *k*_21_ = 6 (A,B) and *k*_21_ = 10 (C,D), with *z*_2_ = *x*_2_ + *iy*_2_**.

The result is multi-frequency periodic behavior in the response system that is more complicated than in the previous example. Figure 6B shows a plot of the *local* minima and maxima of *y*_2_ = Im(*z*_2_) vs. *k*_21_. Figure 7 shows *y*_2_ vs. *x*_2_ plots of the periodic orbits at *k*_21_ = 6 (upper panels) and *k*_21_ = 10 (lower panels). As *k*_21_ increases from zero, a periodic orbit with winding number two emerges (similar to that shown in Figure 7A) and grows in amplitude, peaking near *k*_21_ ≈ 2.5. The amplitude subsequently decreases to a minimum near *k*_21_ ≈ 7.2, and then slowly increases again. Note that the four curves in Figure 6B for *k*_21_ ∈ [0, 7.2] correspond to two pairs of alternating local maxima and minima in the time series of *y*_2_, as shown in Figure 7B.

Interestingly, near *k*_21_ ≈ 7.2, an additional loop appears in the orbit, as shown in Figure 7C. This is reflected in the additional inner curves in Figure 6B that appear for *k*_21_ ⪆ 7.2, and the two additional local maxima and minima in the time series of *y*_2_ in Figure 7D.

#### 4.2.2. Multistability in the response system

Continuing with the excitatorily coupled response system (with *k*_22_ = 9 > 0), we set η_2_ = −10 and leave all other parameters unchanged. In this case the uncoupled response system is at a point just to the left of the left SN curve in Figure 2A, and as *k*_21_ increases, η_*eff*_ again sweeps back and forth along the horizontal line at Δ_1_ = 0.5. However, now this sweeping cuts across both SN curves. Thus, the response system sweeps back and forth across the approximately triangular multistable region bounded by the SN curves.

**Figure 8:**
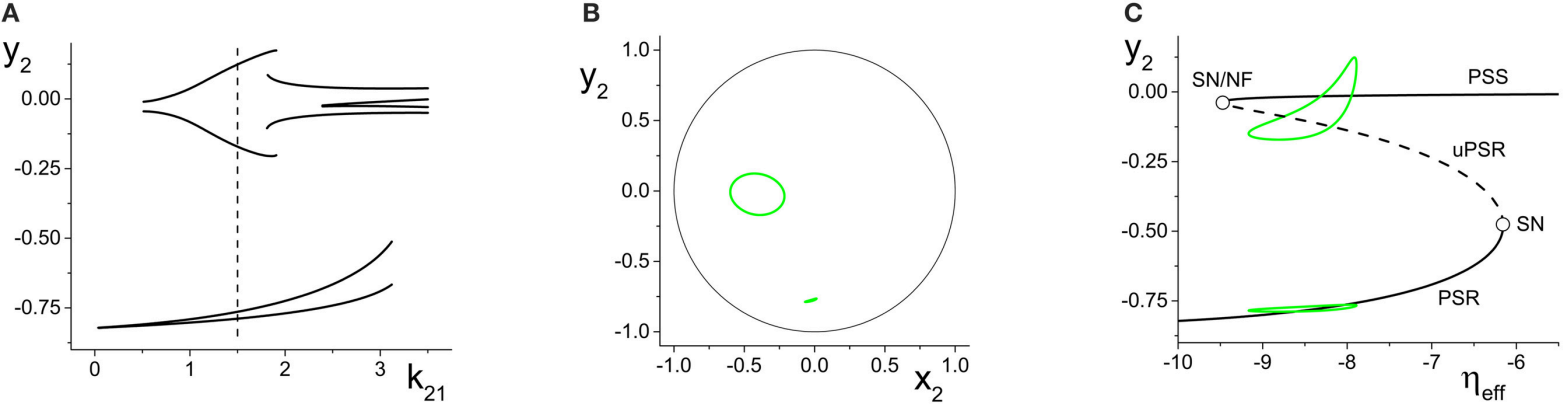
**Multistability in the response system driven by a CPW state of the driver. (A)** Local maxima and minima of *y*_2_ = Re(*z*_2_) vs. the inter-population coupling *k*_21_. **(B)** *y*_2_ vs. *x*_2_ plots showing two coexisting limit cycles of the response system at *k*_21_ = 1.5 (dotted vertical line in **A**). **(C)** The solid and dashed black curves show the asymptotic states of the response for fixed values of η_*eff*_, with *k*_21_ = 1.5. Green curves are coexisting limit cycles of the response system when coupled to the driver. Parameters are: η_1_ = 10.75, Δ_1_ = 0.5, *k*_11_ = −9; η_2_ = −10, Δ_2_ = 0.5, *k*_22_ = 9.

Figure 8A shows the maxima and minima of *y*_2_ vs. *k*_21_ for this case. The first feature to emerge as *k*_21_ increases from zero is a simple periodic orbit whose amplitude increases, similar to the example in Figure 6A. At *k*_21_ ≈ 0.5, a new and separate coexisting limit cycle appears, as indicated by the upper curves that emerge in Figure 8A. Figure 8B shows the *y*_2_ vs. *x*_2_ plots of these two limit cycles at *k*_21_ = 1.5, where the larger orbit corresponds to the upper two curves in Figure 8A. In this bistable region, the macroscopic dynamics of the response system approaches one or the other of these periodic states, depending on the initial conditions.

Figure 8C shows, in black, the asymptotic states of *y*_2_ vs. η_*eff*_ for *fixed* values of η_*eff*_, with *k*_21_ = 1.5. These curves show that for a large interval of η_*eff*_, a stable PSR coexists with a stable PSS and an unstable PSR state for the frozen (i.e., η_*eff*_ fixed) system. With the driver on the CPW state, η_*eff*_ sweeps from approximately −9.1 to −7.6 and back again–a range which is well within the bistable region. Superimposed in green in Figure 8C are projections of the two coexisting limit cycles onto this space, showing that the lower limit cycle is a simple periodic perturbation of the response system's underlying PSR state, and the upper limit cycle is a periodic perturbation of the underlying PSS state.

#### 4.2.3. Chaos in the response system

We now switch to the inhibitorily coupled response system, with parameters η_2_ = 5, Δ_2_ = 0.5, and *k*_22_ = −9. The parameter space of this system is shown in Figure 3A, and the uncoupled response system resides at the solid black dot in that figure, to the left of all the bifurcations. As the interpopulation coupling strength *k*_21_ increases, η_*eff*_ sweeps across the same horizontal line at Δ_2_ = 0.5 with increasing amplitude and centroid, initially crossing just the left SN bifurcation curve. At *k*_21_ ≈ 5.2, η_*eff*_ begins sweeping across the homoclinic and the Andronov-Hopf bifurcation curves. Eventually, for sufficiently large *k*_21_, η_*eff*_ sweeps across all four bifurcation curves (SN, AH, HC, and SN).

**Figure 9:**
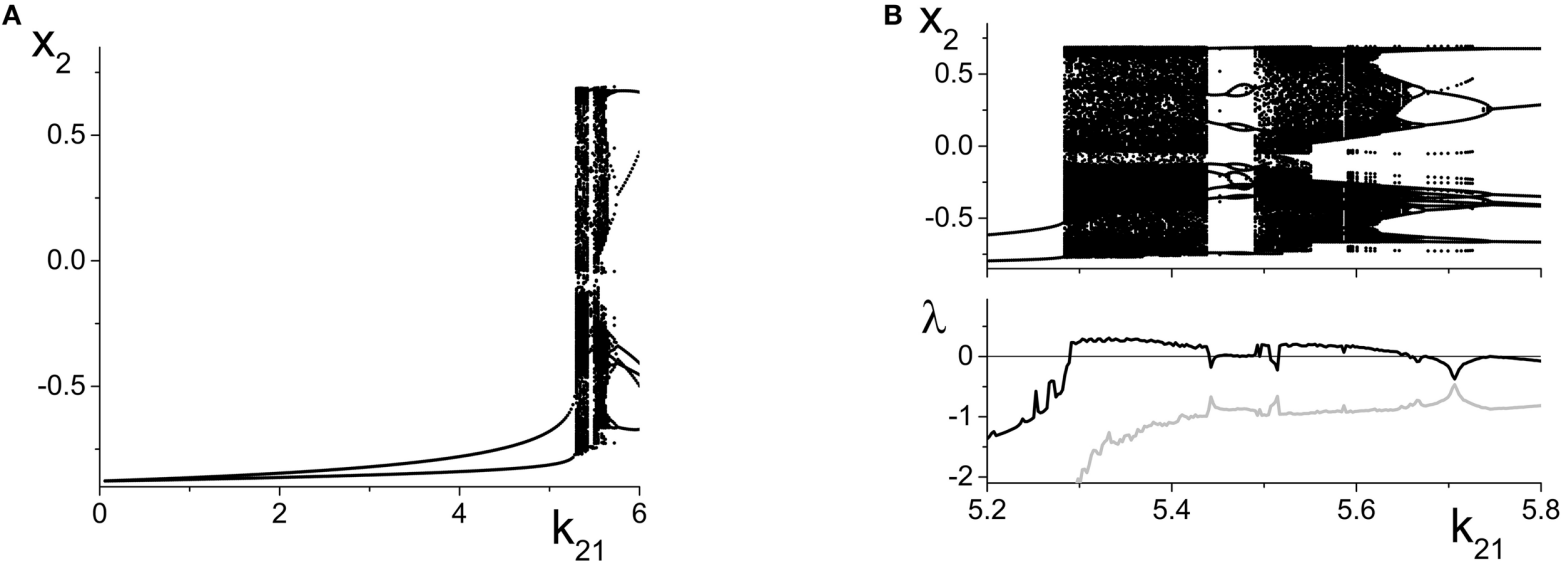
**Emergence of macroscopic chaos in the response system driven by a CPW state of the driver. (A)** Local minima and maxima of *x*_2_ = Re(*z*_2_) vs. the inter-population coupling *k*_21_. **(B)** Magnification of the chaotic region (top), with a plot of the largest two Lyapunov exponents. Parameters are: Δ_1_ = 0.5, *k*_11_ = −9, η_1_ = 10.75; Δ_2_ = 0.5, *k*_22_ = −9, η_2_ = 5.

Figure 9A shows the local maxima and minima of *x*_2_ = Re(*z*_2_) vs. *k*_21_. We initially see the emergence of a simple periodic orbit that grows slowly in amplitude. However, at *k*_21_ ≈ 5.2, chaos suddenly emerges through a crisis. Figure 9B shows a magnification of this region, with a plot of the two largest Lyapunov exponents. We see that there are significant intervals of *k*_21_ for which there is a positive Lyapunov exponent, indicating the presence of macroscopic chaos.

As *k*_21_ increases, the first chaotic band, beginning at *k*_21_ ≈ 5.28, coexists with the simple periodic loop that was present for smaller *k*_21_ (this coexistence is not apparent in the figure). Outside of this band, there is a window dominated by periodic behavior of rather high period. A second chaotic band appears at approximately *k*_21_ = 5.48. This second band terminates at approximately *k*_21_ = 5.65, after which a series of reverse period-doubling cascades are seen.

**Figure 10:**
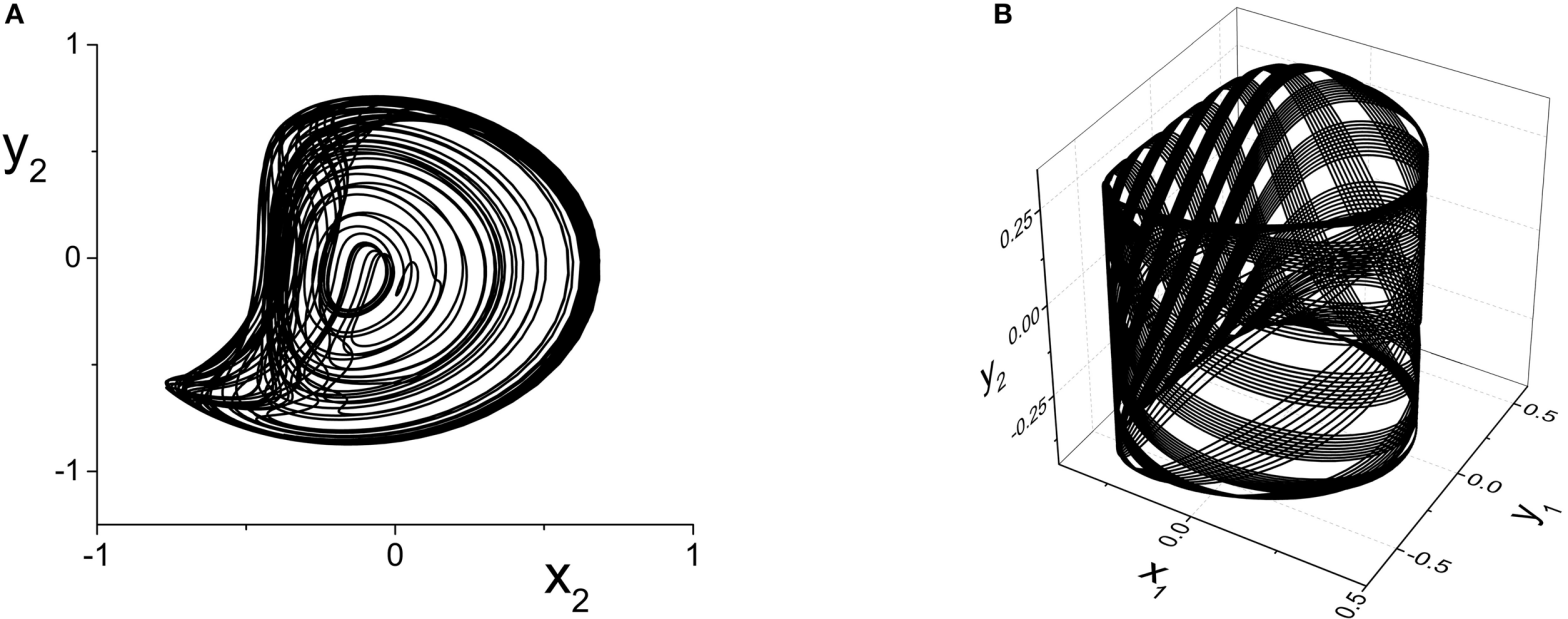
**(A)** Chaotic (*y*_2_ vs. *x*_2_) and **(B)** Quasiperiodic (*y*_2_ vs. *x*_1_ vs. *y*_1_) attractors in the response system driven by a CPW state of the driver. Parameters are: *k*_11_ = k_22_ = −9, with **(A)** η_2_ = 5, Δ_1_ = Δ_2_ = 0.5 and *k*_21_ = 5.296, and **(B)** η_2_ = 10.75, Δ_1_ = 0.5, Δ_2_ = 0.3, and *k*_21_ = 0.1.

The *y*_2_ vs. *x*_2_ plot of the chaotic attractor present at *k*_21_ = 5.296, for which the largest Lyapunov exponent is approximately 0.2118, is shown in Figure 10A.

#### 4.2.4. Quasiperiodicity in the response system

Finally, we consider the case in which the response system exhibits a CPW state when uncoupled from the driver, and ask what happens when this is driven by another CPW state in the driver. We use the same drive system parameters as above, and set the response system's parameters to be the same except for Δ_2_ = 0.3. As the inter-population coupling strength *k*_21_ is increased, various phase-locked and quasiperiodic states are seen. An example of quasiperiodic behavior in the response system for *k*_21_ = 0.1 is shown in Figure 10B.

## 5. Discussion

In this work, we have taken the first step toward designing a mathematically tractable modular network-of-networks representation of neuronal systems. Our approach is based on dynamical analysis techniques that enable a complete description of the emergent macroscopic behavior of large, heterogeneous discrete networks of globally-coupled phase oscillators. Building on previous results (Luke et al., 2013) in which we used these techniques to show that the collective dynamics of a single such population of theta neurons is relatively simple (exhibiting just equilibria and limit cycle states), we constructed the next simplest hierarchical structure: a driver-response configuration of theta neuron populations. Our results show that even in this simplest of configurations, the response system (and hence, the network as a whole) can exhibit a full range of dynamical behaviors and surprising complexity. A notable strength of our work is that despite the complexity that emerges from this arrangement, the behavior can be understood and explained in terms of what is known about a single population's dynamics and bifurcation structure.

With the driving system on a fixed equilibrium, we showed that the response system is equivalent to a single population with a simple shift in one parameter. Specifically, this parameter is the median of the distribution of excitability parameters in the response system, which indicates whether the response population is dominated by excitable or intrinsically-spiking neurons. Although this arrangement does not introduce any new dynamical features, we showed that the response system can nevertheless still exhibit an interesting bifurcation structure involving macroscopic equilibria, limit cycles, and multistability as the strength of the inter-population coupling varies. More interestingly, we found that the inter-population coupling strength is effectively equivalent to the response system's median intra-population excitability. By this we mean that changes in either of these rather different network parameters lead to identical bifurcation scenarios. This surprising result follows from the drive-response network configuration in particular.

The first level of additional complication arose when modestly altering an internal parameter of the drive system. This effectively led to a *non-linear* change in the response system's median excitability, causing a dramatic change in the response's bifurcation structure. Such bifurcation structures might be difficult to understand if encountered blindly, as might be the case when studying the dynamics of a network without knowledge of its internal structure. Experimental studies of neuronal networks often take a similar “black box” approach out of necessity, since detailed knowledge of connectivity (i.e., the “connectome”) is rarely available. In our case, however, we showed that knowledge of the non-linearity, along with knowledge of the bifurcation structure of a single network, leads to a natural explanation of the additional features that arise due to the network-of-networks structure. In our particular case studies, we observed multiple distorted and reversed copies of the bifurcation structure that is associated with a single population of theta neurons. We therefore speculate that in “black box” investigations, the observation of such repeated and/or distorted bifurcation structures might be indicative of driver-response-type connectivity in the network of study.

Finally, we investigated the consequences of placing the driver system on a collective rhythmic state (i.e., a macroscopic periodic orbit). Our results were consistent with previous results that studied non-autonomous phase oscillator (So and Barreto, 2011) and theta neuron systems (So et al., 2014). In those investigations, it was shown that networks of oscillators subjected to a sinusoidal variation of a network parameter led to complicated dynamics including quasiperiodicity and macroscopic chaos. Here, our driver-response arrangement of two separate interacting populations of theta neurons leads to an overall autonomous system, but with the response system being subjected to a periodic driving signal from the driver. Such arrangements might be found in real neuronal systems at the early stages of sensory input processing. For example, the lateral geniculate nucleus may be driven by a periodic visual signal delivered to the retina. Another candidate might be the trisynaptic circuit of the dentate gyrus and the CA3 and CA1 regions of the hippocampus (Kandel et al., 2000). More generally, the information-processing capabilities of the brain are thought to be regulated by collective rhythms, notably theta and gamma oscillations, which arise in various areas and periodically drive other areas (Buzsáki, 2006).

Our results may also have implications for populations of bursting neurons (So et al., 2014). Neuronal bursting in individual neurons is commonly understood to arise as the result of the interplay between a slowly oscillating neuronal parameter (or “slow variable”) and the neuron's fast spiking dynamics. Bursting arises if the slow parameter sweeps back and forth across bifurcations, and (Rinzel and Ermentrout, 1989) classified bursters as square, parabolic, or elliptic based on the bifurcations encountered in this process. It has also been demonstrated that slowly oscillating intra- and extra-cellular ion concentrations can lead to wide range of neuronal bursting behaviors (Cressman et al., 2009, 2011; Barreto and Cressman, 2011).

Finally, we note that our explorations in this work were limited to cases in which the driver population's parameters were either fixed or were varied only modestly. In the latter case, we changed the driver's median excitability parameter only to the extent that its collective equilibrium state was displaced but not altered. Significantly greater complexity in the response's dynamics would arise if the collective state of the driver were pushed across its own bifurcations, possibly resulting in topological changes and hysteretic effects in the driver's macroscopic state. As discussed above, such complexity would be difficult to understand if encountered in a “black box”-type investigation. Nevertheless, if it is known that the network of interest has a driver-response structure, it may be possible to comprehend the origin of such complexity in the manner that we have outlined here.

This study constitutes an initial attempt at building a mathematically tractable model to understand the collective behavior of a hierarchical “network-of-networks” arrangement of model neurons. In future work we plan to consider networks of networks that include feedback connections and additional populations in an effort to understand the emergence of macroscopic dynamical complexity in more realistic networks.

## Author contributions

Tanushree B. Luke, Ernest Barreto, and Paul So conceived and designed the investigation, analyzed the data, and wrote the paper. Tanushree B. Luke and Paul So performed the numerical computations.

### Conflict of interest statement

The authors declare that the research was conducted in the absence of any commercial or financial relationships that could be construed as a potential conflict of interest.

## Footnotes

^1^$a_{n}=2\pi /\int_{-\pi }^{\pi }{\left(1-\cos(x)\right)}^{n}=n!/(2n-1)!!$
